# A Seven‐Step Framework for Structured ChatGPT‐Assisted Pharmacology Learning: Educational Outcomes and Attitudes Toward Hypothetical AI‐Informed Patients

**DOI:** 10.1155/sci5/3725461

**Published:** 2026-10-22

**Authors:** Jelena Roganović, Igor Končar, Milena Barać

**Affiliations:** ^1^ Department of Pharmacology in Dentistry School of Dental Medicine University of Belgrade Belgrade Serbia bg.ac.rs; ^2^ Clinic for Vascular and Endovascular Surgery of the University Serbian Clinical Center Belgrade Serbia

**Keywords:** AI-informed patients, ChatGPT, clinical reasoning, generative artificial intelligence, healthcare education, patient communication, pharmacology education

## Abstract

**Background/Objectives:**

Generative artificial intelligence is increasingly used by health professions students and by patients seeking health information before consultation. This creates two related educational challenges: supporting critical AI use during learning and preparing future clinicians to respond appropriately to hypothetical AI‐informed patients. This study evaluated a teacher‐designed seven‐step ChatGPT‐assisted framework for autonomic pharmacology learning and explored whether principles embedded in the framework were reflected in students’ attitudes toward AI‐informed patients.

**Methods:**

In this matched‐cohort educational study, 104 third‐year dental students were included. Students who voluntarily participated in the seven‐step ChatGPT‐assisted framework (*n* = 52) were individually matched with nonusers (*n* = 52) by previous academic achievement and digital‐tool familiarity. The primary outcome was performance on a three‐item open‐ended pharmacology quiz assessing mechanistic reasoning, scored independently by two blinded assessors. Framework participants also completed an eight‐item learning‐perception questionnaire and a ten‐item questionnaire addressing hypothetical AI‐informed patients. Exploratory Spearman correlations examined conceptually matched learning‐ and patient‐facing statements.

**Results:**

Framework participants achieved higher postlearning pharmacology scores than matched nonusers (1.77 ± 0.30 vs. 1.45 ± 0.47; *p* = 0.0003). The largest difference was observed for questions requiring integration of receptor mechanisms and downstream signaling (+28%; *p* < 0.001), whereas factual drug‐name recall did not differ (*p* = 0.47). Students generally recognized both the potential usefulness and limitations of ChatGPT and endorsed professional, nondismissive responses to hypothetical AI‐informed patients. The perceived usefulness of ChatGPT for learning and writing was associated with recognition of the risk of patient overreliance on AI‐generated diagnoses (*ρ* = 0.32, *p* = 0.019), while intention to use ChatGPT for learning was associated with endorsement of a serious and professional approach to patients presenting with ChatGPT‐generated diagnoses (*ρ* = 0.40, *p* = 0.004).

**Conclusions:**

Participation in the structured framework was associated with stronger postlearning mechanistic pharmacology performance. The novel cross‐context analysis identified limited but potentially meaningful alignment between responsible AI use in learning and professional attitudes toward hypothetical AI‐informed patients. These exploratory associations do not demonstrate transfer of clinical communication competence, but suggest that verification, contextualization, risk awareness, and human oversight may provide a shared educational foundation for addressing AI use by both learners and patients.

## 1. Introduction

Generative artificial intelligence (AI), including conversational systems such as ChatGPT, is increasingly used by health‐professions students and patients. In education, these tools can support iterative questioning, rapid explanation, and personalized feedback. However, fluent outputs may contain factual inaccuracies, fabricated references, or oversimplified reasoning, while unstructured use may encourage overreliance and passive acceptance of generated content [1–3]. The educational challenge is therefore to structure AI use so that it supports, rather than replaces, disciplinary reasoning. This challenge is particularly relevant to autonomic pharmacology. The autonomic nervous system regulates cardiovascular function, airway tone, gastrointestinal motility, glandular secretion, pupillary responses, bladder function, and physiological responses to stress. Drugs acting on adrenergic and cholinergic receptors are consequently used across anesthesia, emergency medicine, cardiology, respiratory care, ophthalmology, dentistry, surgery, nursing, and pharmacy practice. Understanding receptor selectivity, intracellular signaling, and tissue‐specific effects is necessary not only for selecting appropriate treatment but also for anticipating adverse reactions, contraindications, and clinically important drug interactions. In dentistry, autonomic pharmacology is relevant to the safe administration of local an aesthetics containing vasoconstrictors, the management of xerostomia and salivary dysfunction, the recognition of autonomic adverse effects of commonly used medicines, and the treatment of medically complex patients receiving antihypertensives, bronchodilators, antidepressants, or anticholinergic drugs. In medicine and nursing, this knowledge supports the management of hypotension, arrhythmias, bronchospasm, anaphylaxis, perioperative instability, and toxicological emergencies. Conversational AI may support this process, but its value depends on the quality of prompting, prior knowledge, expectation calibration, and independent verification [4]. Teacher‐guided approaches may preserve learner responsibility by combining defined objectives, critical questioning, verification, active synthesis, and reflection.

Critical appraisal of AI is also becoming relevant to patient care. Patients increasingly use conversational AI to obtain preliminary explanations, diagnostic labels, and lists of drugs used before consulting healthcare professionals. Although AI systems may improve access to general information, they cannot perform a clinical examination, establish diagnostic validity, or fully contextualize an individual history [5, 6]. AI‐generated interpretations may consequently promote excessive trust, anxiety, narrative narrowing, or expectations that complicate clinical communication. Existing healthcare and dental AI frameworks emphasize transparency, accountability, professional responsibility, and meaningful human oversight [7, 8]. These responsibilities apply not only when clinicians use AI directly, but also when they manage the consequences of AI use initiated independently by patients. Thus, AI‐assisted learning and hypothetical encounters with AI‐informed patients may therefore share several principles: verification, contextualization, recognition of uncertainty, risk awareness, and human oversight.

Research in health‐professions education has largely examined AI performance, usability, perceptions, and general educational applications [9, 10]. Fewer studies have evaluated structured AI‐assisted learning alongside students’ attitudes toward patients whose beliefs have been shaped by conversational AI. The primary aim of this study was to determine whether participation in a teacher‐designed seven‐step ChatGPT‐assisted framework was associated with postlearning conceptual understanding and mechanistic reasoning in autonomic pharmacology. Secondary aims were to describe students’ perceptions of ChatGPT‐supported learning and their attitudes toward hypothetical AI‐informed patient encounters and to examine conceptually matched associations between learning‐related and patient‐facing attitudes.

## 2. Materials and Methods

### 2.1. Study Design and Setting

This matched‐cohort educational study evaluated a teacher‐designed seven‐step ChatGPT‐assisted framework in third‐year dental pharmacology students at the School of Dental Medicine, University of Belgrade (*n* = 104) enrolled in the Pharmacology in Dentistry course during the 2023–2024 academic year. The sample size was determined by the number of eligible third‐year dental students enrolled in the course during the study period, and all 104 were included in the study. Ethical approval was obtained from the institutional review board (Ethical Committee of the School of Dental Medicine, approval No. 36/50, 2023). The study examined three levels of outcome: objective pharmacology performance, students’ perceptions of ChatGPT‐supported learning, and ChatGPT‐informed patient‐facing outcomes. Students voluntarily indicated whether they would use freely available online ChatGPT‐3.5 in their studies. Based on this choice, they participated in either a ChatGPT‐framework group (*n* = 52) or a non‐ChatGPT‐framework group (*n* = 52), which used usual literature (Pharmacology book). To reduce bias, each ChatGPT user was matched with a nonuser based on two baseline variables: average academic grade (±0.5 grade points) and digital tool familiarity (±1 point on a three‐point Likert scale). In instances where multiple nonusers met the exact matching criteria for a single framework participant, the final control match was selected using a random number generator to prevent selection bias. However, residual confounding related to motivation, prior AI experience, learning strategies, and time on task could not be excluded. With 52 matched pairs, the study had sufficient sensitivity to detect a moderate mean difference of 0.40 at a two‐sided α of 0.05% and 80% power.

### 2.2. Seven‐Step ChatGPT‐Assisted Educational Framework

The teacher‐designed framework was developed for learning autonomic pharmacology and combined structured AI use with active learning activities. Its seven steps were as follows (Figure 1):

1. Teacher pretesting: The instructor pretested ChatGPT responses to the selected pharmacology topics and identified potential factual errors, omissions, and misleading explanations. This analysis directly informed the design of the learning milestones and checkpoints in Step 3.
2. Expectation calibration: Students were introduced to ChatGPT’s functions, potential educational value, and limitations.
3. Guided learning structure: Utilizing insights from Step 1, the instructor defined learning objectives, milestones, and reflective checkpoints without providing ready‐made answers.
4. Student‐generated prompting: Utilizing prompt engineering techniques, students formulated prompts and follow‐up questions concerning receptors, signaling pathways, agonists, antagonists, and tissue‐specific drug effects.
5. Independent verification: Students checked AI‐generated information against at least two reliable scientific or peer‐reviewed sources, specifically targeting the recognition and mitigation of AI hallucinations.
6. Active visual synthesis: Students translated the information into hand‐drawn pharmacological schemes connecting receptor mechanisms, intracellular pathways, drug actions, and clinical implications. This step served strictly as a formative cognitive engagement tool for multimodal active synthesis, not as a summative assessment instrument.
7. Reflective discussion: Students discussed the usefulness, limitations, risks, and responsible use of ChatGPT during a concluding seminar.

**FIGURE 1:**
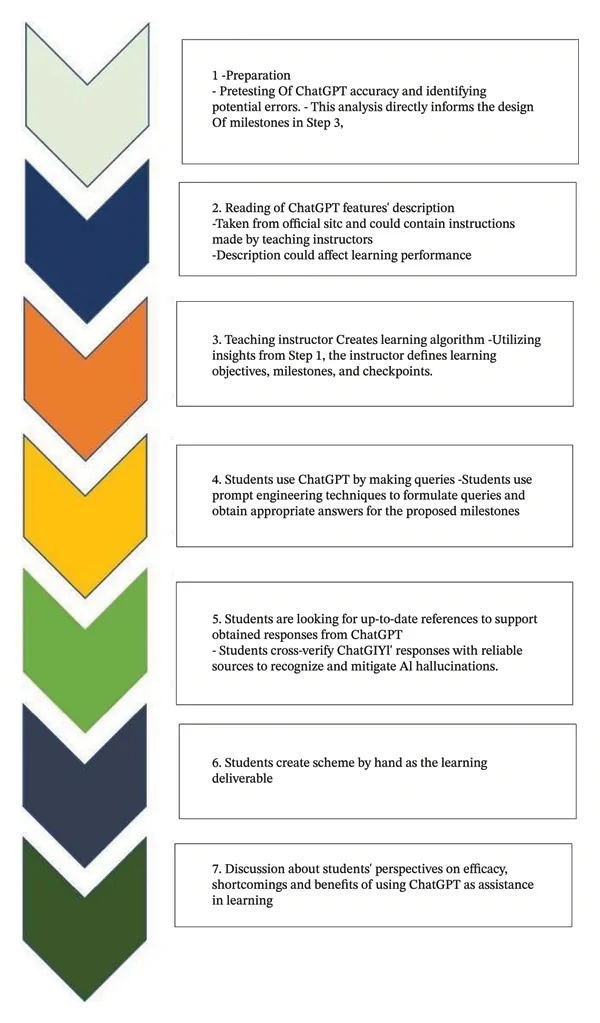
Seven‐step framework for structured ChatGPT‐assisted pharmacology learning.

The seven steps were selected to operationalize the pedagogical principles of guided discovery, active cognitive engagement, independent verification, multimodal synthesis, reflection, and instructor oversight. The framework was not intended as an exhaustive taxonomy of human–AI interaction skills, but as a feasible sequence tailored to the specific autonomic pharmacology learning activity. Constructivist learning, guided discovery, cognitive engagement, multimodal representation, and reflective learning informed the design and sequencing of the framework [11–13]. These theories served as a design rationale and were not tested as mediators of the observed outcomes (Table 1).

**TABLE 1:** Conceptual foundations of the seven‐step ChatGPT‐assisted learning framework and their hypothesized relevance for AI‐informed patient encounters.

Theoretical rationale	Key principle	Framework activity	Intended learning process	Hypothesized relevance for patient encounters
Piaget (1964)–Constructivism	Students learn best when they connect new information with prior knowledge and actively reconstruct understanding through experience.	Students formulate prompts to explore pharmacological mechanisms and connect ChatGPT‐generated explanations with previously learned biomedical principles.	Prompt‐dependent reasoning and cognitive integration.	Patients also generate AI responses through their own symptom descriptions and prompts.
Jonassen (1991)–Guided Discovery	Learning occurs through structured exploration rather than passive reception of ready‐made answers.	The instructor provides a learning algorithm with milestones, reflective checkpoints, and goals, but does not provide ready‐made answers.	Guided autonomy and professional oversight.	Dentists must not simply dismiss or accept AI‐generated patient beliefs. They must guide patients back toward clinical examination, professional judgment, and shared understanding.
Cognitive Engagement Theory	Learning improves when students actively question, verify, transform, and reflect on information.	Students cross‐verify ChatGPT responses with peer‐reviewed literature and produce hand‐drawn pharmacological diagrams.	Verification, risk awareness, and translation of AI text into professional reasoning.	Patients may trust fluent AI explanations without verification. Dentists must explain AI limitations and correct misinformation.
Reflective and ethical learning	Professional competence requires awareness of benefits, limitations, uncertainty, and responsibility.	Students discuss the efficacy, shortcomings, and benefits of ChatGPT‐assisted learning.	Ethical and patient‐centered mediation.	Dentists must respond seriously and empathically to patients with AI‐generated diagnoses, preserving ethical principles, trust, and human oversight.

### 2.3. The Learning Outcomes

The primary outcome was performance on a postlearning pharmacology quiz consisting of three open‐ended questions on autonomic pharmacology. The open‐response questions used short pharmacological scenarios requiring students to identify the relevant muscarinic receptor subtype, describe its G‐protein and second‐messenger pathway, and use this information to explain or predict tissue‐level effects (e.g., explain how muscarinic stimulation can produce vasodilation even though an increase in intracellular Ca^2+^ usually causes smooth‐muscle contraction; How acetylcholine can decrease heart rate while simultaneously increasing salivary secretion?). The questions assessed conceptual accuracy and the quality of pharmacological reasoning, including students’ ability to connect receptor selectivity, intracellular signaling, and tissue‐level effects. Each response was independently scored by two assessors who were blinded to group allocation, using a predefined analytic rubric: 0 = incorrect or missing response; 1 = partially correct response or incomplete reasoning; 2 = complete and accurate response demonstrating coherent reasoning. A participant‐level quiz score was calculated as the mean of the three item scores, with a possible range from 0 to 2. The quiz questions and analytic rubric were developed by pharmacology teachers and mapped to the learning objectives of the autonomic pharmacology activity. The analytic rubric ensured transparent, criterion‐based evaluation, and inter‐rater reliability was high (ICC = 0.89, 95% CI 0.83–0.93). Because the quiz was administered only after the learning activity, the outcome represented a postlearning between‐group difference rather than demonstrated improvement from baseline. The quiz was also topic‐specific and short; it was not intended to measure long‐term retention, prescribing competence, or clinical performance.

Seminar performance was examined as a secondary educational outcome. Scores from students participating in the 2023–2024 course were compared with those of a historical 2022 cohort who had completed the same pharmacology seminar without participation in a ChatGPT‐assisted framework. The seminar instructions, time limits, assessment criteria, and permitted resources were kept as similar as possible across cohorts. Nevertheless, because the comparison involved different academic years, possible cohort effects could not be excluded. Third, following the learning activity, students from the ChatGPT‐assisted framework group completed an eight‐item questionnaire concerning their perceptions of ChatGPT‐supported learning. Responses were recorded on a five‐point Likert scale ranging from 1, *strongly disagree*, to 5, *strongly agree*. The items addressed perceived usefulness for learning; perceived speed of learning; ease of use and engagement; intention to continue using ChatGPT; general acceptance of its use; and perceived educational risk, and were retained verbatim from the previously published 11‐item questionnaire [4] because they directly addressed the constructs relevant to the present intervention. The eight‐item learning‐perception questionnaire demonstrated good internal consistency (Cronbach’s α = 0.799).

### 2.4. Questionnaire on Attitudes Toward Hypothetical AI‐Informed Patients

A ten‐item questionnaire examined students’ attitudes toward hypothetical encounters with patients who had consulted ChatGPT for orofacial self‐diagnosis before seeking professional care. Prior to data collection, the questionnaire underwent face and content validation by a panel of three independent experts in pharmacology and dental education to ensure item clarity, clinical relevance, and construct representativeness. Items were rated on the same five‐point Likert scale. The questionnaire addressed: the limited informational role of ChatGPT before a dental visit; the possibility that fluent responses may be clinically inaccurate; the inability of ChatGPT to replace clinical examination; the risk of patient overtrust; potential effects on the dentist–patient relationship; the professional responsibility to explain AI limitations; endorsement of serious, calm, and empathic responses to patients. The ten‐item patient‐interaction questionnaire showed good internal consistency (Cronbach’s α = 0.834). Given the sample size, this structure was considered exploratory and was not interpreted as formal factorial validation.

### 2.5. Cross‐Context Survey Analysis

To explore whether conceptually similar attitudes toward AI were expressed across learning and patient‐facing contexts, an exploratory cross‐context item‐level analysis was performed. The conceptual pairings were established a priori during the study design phase. The selected statements from the ChatGPT learning survey were paired with conceptually corresponding statements from the hypothetical AI‐informed patient survey according to shared themes: perceived usefulness of AI, critical awareness of AI limitations, responsible AI adoption and overreliance, professional education about AI limitations, constructive professional engagement with AI, professional and relational risks of AI, explanation and contextualization of AI‐generated information, and AI‐related risk awareness with supportive communication. The association between the items was examined using Spearman’s rank‐order correlation.

### 2.6. Statistical Analysis

Continuous outcomes were presented as means and standard deviations, while Likert‐scale items were reported using frequencies and percentages. Pharmacology quiz scores were compared between individually matched framework participants and nonusers using a paired‐samples *t*‐test.

Seminar scores from different academic cohorts were treated as independent observations and compared using an unpaired *t*‐test. Multiple linear regression was used to examine the association between participation in the ChatGPT‐assisted framework and seminar grade while adjusting for cohort, gender, and attendance. Results were reported as unstandardized coefficients (B) with 95% confidence intervals.

Spearman’s rank‐order correlation was used for cross‐context item‐level analysis. All tests were two‐sided, with statistical significance set at *p* < 0.05. Analyses were conducted using GraphPad Prism Version 11.

## 3. Results

### 3.1. Learning Outcomes After Structured ChatGPT‐Assisted Pharmacology Learning

Students who participated in the ChatGPT‐assisted framework achieved higher mean quiz scores than matched nonusers (1.77 ± 0.30 vs 1.45 ± 0.47; *p* = 0.0003). Figure 2a). The most significant difference was observed in answers requiring integration of receptor mechanisms and downstream signaling pathways (mean = + 28%, *p* < 0.001). In contrast, factual recall of drug names showed no statistical difference between groups (*p* = 0.47). As a secondary outcome, students from the 2023–2024 cohort achieved higher end‐of‐course seminar scores than students from the historical 2022–2023 cohort (1.88 ± 0.64 vs. 1.68 ± 0.56, *p* = 0.02; Figure 2b). Participation in the ChatGPT‐assisted framework remained associated with a higher seminar grade after adjustment for cohort, gender, and attendance (*B* = 0.70, 95% CI 0.55–0.86, *p* < 0.0001; Table 2).

**FIGURE 2:**
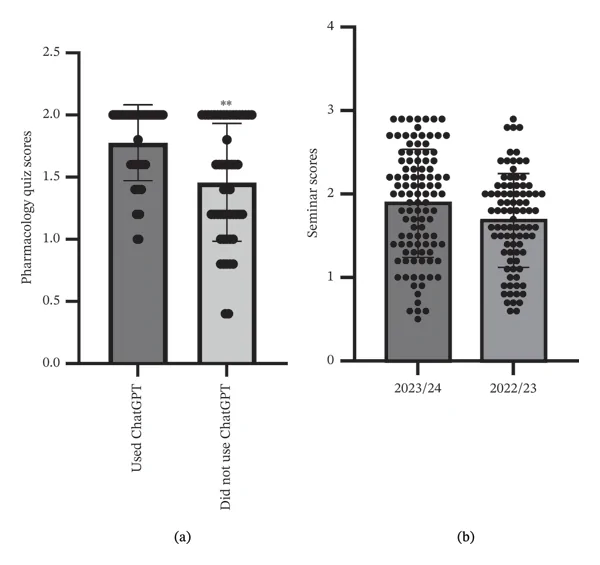
Pharmacology quiz and seminar performance associated with participation in the seven‐step ChatGPT‐assisted framework. The bars represent mean ± SD of quiz (a) and seminar scores (b). Quiz scores differed between groups (*p* = 0.0003), while seminar scores differed between cohorts (*p* = 0.02).

**TABLE 2:** Adjusted association between participation in the ChatGPT‐assisted framework and seminar performance.

Dependent variable	Independent variables	Estimates	95% CI	*p* value
Seminar grades	ChatGPT‐framework participation	0.7037	0.5457 to 0.8616	< 0.0001
	Group (2022 vs. 2023)	−0.1923	−0.3322 to −0.05235	0.0073
	Gender	0.1981	0.05872 to 0.3375	0.0056
	Attendance	0.1103	−0.1639 to 0.3845	0.4285

### 3.2. Learning‐Related Perceptions of ChatGPT

After structured ChatGPT‐assisted learning, students perceived ChatGPT as easy to use and potentially useful, but their responses also reflected uncertainty regarding its educational value and appropriateness as a learning tool (Figure 3).

**FIGURE 3:**
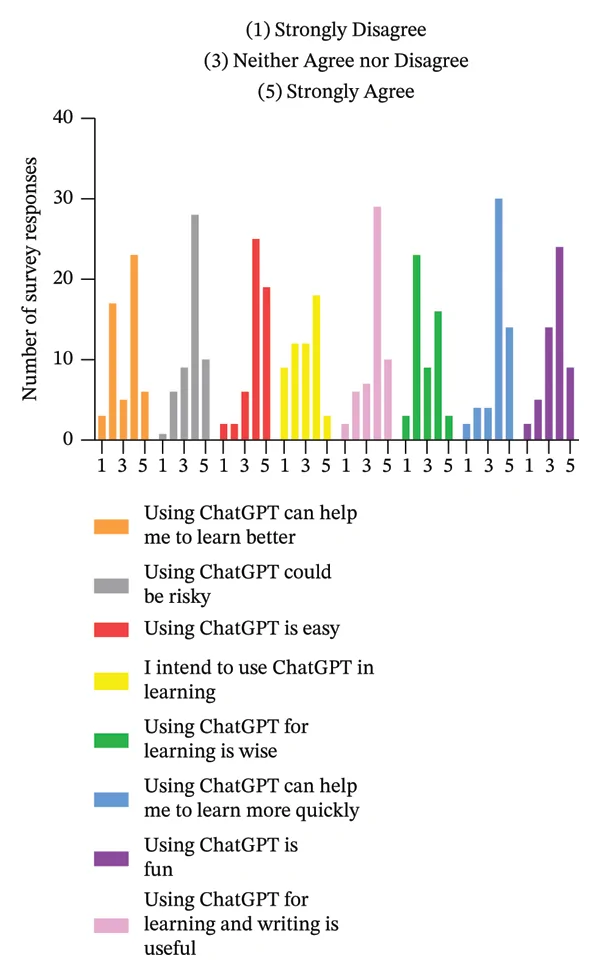
Students’ perceptions of using ChatGPT for pharmacology learning. Responses were rated on a Likert scale from 1 (*strongly disagree*) to 5 (*strongly agree*). Statements included aspects of usefulness, risks, ease of use, intention to use, and enjoyment.

### 3.3. Perceptions of Hypothetical AI‐Informed Patients

In the patient‐interaction survey, students similarly recognized ChatGPT’s limited informational value while strongly rejecting its ability to replace clinical examination. Most students agreed that ChatGPT‐generated answers may sound convincing despite lacking clinical accuracy, that patients may place excessive trust in AI‐generated diagnoses, and that dentists should explain the limitations of AI tools. Students also emphasized the need for a serious, professional, calm, and empathic response to patients presenting with AI‐generated diagnostic beliefs (Figure 4).

**FIGURE 4:**
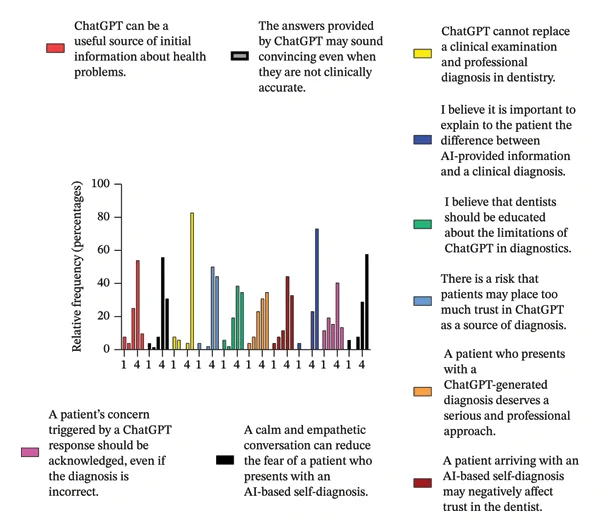
Students’ perceptions of hypothetical AI‐informed patients. Responses were rated on a Likert scale from 1 (*strongly disagree*) to 5 (*strongly agree*).

### 3.4. Cross‐Context Analysis

An exploratory cross‐context analysis was conducted among the 52 students with linked learning‐perception and patient‐interaction responses. Two conceptually matched cross‐context associations were statistically significant (Table 3).

**TABLE 3:** Conceptually matched associations between ChatGPT learning and AI‐Informed patient statements.

Cross‐context concept	ChatGPT learning statement	AI‐informed patient statement	Conceptual rationale	Spearman *ρ*	*p*
Perceived usefulness of AI	Using ChatGPT can help me to learn better.	ChatGPT can be a useful source of initial information about health problems.	Both statements assess whether ChatGPT is perceived as a potentially useful informational resource.	0.11	0.429
Critical awareness of AI limitations	Using ChatGPT could be risky.	The answers provided by ChatGPT may sound convincing even when they are not clinically accurate.	Both address the possibility that AI output may be unreliable and therefore require critical evaluation.	0.07	0.644
Responsible AI adoption and overreliance	Using ChatGPT for learning and writing could be useful.	There is a risk that patients may place too much trust in ChatGPT as a source of diagnosis.	Both statements advocate for balanced AI adoption	0.32	0.019
Professional education about AI limitations	Using ChatGPT for learning and writing could be useful.	Dentists should be educated about the limitations of ChatGPT in diagnostics.	Both relate to incorporating AI into professional education while maintaining awareness of its limitations.	0.18	0.204
Constructive professional engagement with AI	I intend to use ChatGPT in learning.	A patient who presents with a ChatGPT‐generated diagnosis deserves a serious and professional approach.	Both reflect willingness to engage constructively with AI‐related situations rather than rejecting or dismissing them.	0.40	0.004
Professional and relational risks of AI	Using ChatGPT could be risky.	A patient arriving with an AI‐based self‐diagnosis may negatively affect trust in the dentist.	Both statements reflect awareness that AI use carries potential risks beyond technical accuracy, including unintended effects on trust and the clinician–patient relationship.	−0.19	0.189
Explanation and contextualization of AI information	Using ChatGPT for learning and writing could be useful.	It is important to explain to the patient the difference between AI‐provided information and a clinical diagnosis.	Both concern using AI‐derived information in a context that requires interpretation, qualification, and professional contextualization.	0.10	0.487
AI‐related risk awareness and supportive communication	Using ChatGPT could be risky.	A calm and empathetic conversation can reduce the fear of a patient who presents with an AI‐based self‐diagnosis.	The pairing examines whether general awareness of ChatGPT‐related risk is associated with support for an empathetic response to patient anxiety prompted by AI‐generated self‐diagnosis.	0.24	0.093

## 4. Discussion

This study evaluated a teacher‐designed seven‐step framework that combined structured ChatGPT use with verification, active synthesis, and reflection. Participation was associated with higher postlearning pharmacology scores, with the largest difference observed for questions requiring integration of receptor mechanisms and intracellular signaling, whereas factual drug‐name recall did not differ. These findings indicate that the framework was associated with stronger performance in mechanistic reasoning rather than simple recall. Because the study was nonrandomized and the intervention included several components, the results should be interpreted as an association with the complete educational framework rather than as an isolated effect of ChatGPT.

A novelty of the study is the examination of AI‐assisted learning together with students’ attitudes toward patients who had independently used conversational AI for self‐diagnosis. This allowed the analysis to move beyond educational performance and examine whether selected principles embedded in the learning framework were also reflected in patient‐facing attitudes. The results support partial conceptual alignment between the two contexts, particularly around verification, contextualization, risk awareness, and human oversight, without demonstrating transfer of clinical communication competence.

Verification was an explicit component of the intervention. Students were required to compare ChatGPT‐generated explanations with reliable scientific sources before using them in pharmacological reasoning. This requirement is relevant because fluent AI‐generated responses may appear credible despite containing inaccurate, incomplete, or fabricated information [1, 14]. The patient‐interaction findings showed a similar pattern: students generally recognized that ChatGPT‐generated health information may sound convincing despite lacking clinical accuracy and that AI cannot replace clinical examination. These findings are consistent with previous concerns about persuasive AI‐generated misinformation [5] and with ethical frameworks emphasizing transparency, accountability, and human oversight in dental AI [7]. In the present study, verification was therefore treated as a learner responsibility rather than as an optional safeguard.

Contextualization was also central to the framework. Students were asked to connect receptors, signaling pathways, drug actions, and tissue‐level effects and to reorganize these relationships in hand‐drawn schemes. The stronger performance in questions requiring mechanistic integration is consistent with this emphasis, although the contribution of the visual synthesis step cannot be separated from the other elements of the intervention. Evidence that handwriting and drawing may engage broader neural networks than typing provides a rationale for including such visuomotor activities [13], but the present study did not test this mechanism directly.

In patient care, contextualization involves placing AI‐derived information within the patient’s symptoms, medication use, chronology, examination findings, concerns, and own description of the problem. This is relevant because conversational AI may reduce complex experiences to apparently precise diagnostic labels while omitting clinically important context [15]. The present patient‐facing findings support the importance of distinguishing AI‐generated interpretations from clinically established conclusions, but they do not show that students could perform this contextualization effectively in real consultations.

Students also appeared to distinguish perceived usefulness from uncritical reliance. They generally accepted that ChatGPT may provide useful preliminary information while recognizing risks related to inaccurate responses, excessive trust, anxiety, and disruption of the clinician–patient relationship. These concerns are consistent with previous reports of misinformation, dependency, reduced independent reasoning, opacity, and overreliance in educational and healthcare settings [2, 3, 6, 16–18]. The framework addressed these risks by retaining responsibility with both the teacher and the learner rather than positioning ChatGPT as an autonomous source of answers.

The patient‐facing results similarly reinforced the importance of professional judgment. Conversational AI cannot perform a physical examination, establish diagnostic validity, or fully contextualize an individual clinical history [5, 6]. Previous work has also raised concerns about dependency, simulated empathy, autonomy, and professional responsibility [19], as well as emotional reliance on conversational systems that lack the accountability and continuity of human care [14, 16–18]. These considerations support the role of meaningful human oversight [7, 8, 20], which was also the most consistent principle embedded in the present framework. Teachers defined objectives and checkpoints and pretested AI responses, while students remained responsible for verification, interpretation, and synthesis. In patient‐facing scenarios, responsibility similarly returned to clinical examination, professional judgment, and patient‐centered dialog.

The exploratory cross‐context correlations provide limited but relevant empirical support for this conceptual connection. Most associations were weak and nonsignificant, indicating that positive perceptions of AI‐assisted learning did not systematically correspond to patient‐facing professional attitudes. The lack of significant correlations for several a priori paired items suggests that learning‐related attitudes toward AI do not consistently align with patient‐facing attitudes. Because the study assessed self‐reported perceptions rather than clinical communication performance, these findings cannot be interpreted as evidence for or against transfer of competence. However, perceived usefulness of ChatGPT for learning and writing was positively associated with recognition of the risk that patients may place excessive trust in AI‐generated diagnoses (*p* = 0.019). This result is consistent with responsible AI adoption, in which recognition of usefulness coexists with awareness of overreliance [1, 7, 20, 21]. In addition, intention to use ChatGPT in learning was associated with endorsement of a serious and professional response to patients presenting with ChatGPT‐generated diagnoses (*p* = 0.004). This was the strongest cross‐context association and suggests that willingness to engage with AI educationally may coexist with a nondismissive professional attitude toward AI‐informed patients.

Thus, these results indicate that selected principles of responsible AI use may be relevant across both educational and patient‐facing settings; however, patient care additionally requires empathy, explanation of uncertainty, negotiation, and preservation of trust, which require explicit communication training.

Several limitations constrain interpretation. Students self‐selected participation, and matching on prior academic achievement and digital‐tool familiarity could not eliminate differences in motivation, prior AI experience, learning strategies, or time on task. Additionally, the study evaluated the framework quantitatively as a holistic educational intervention. Consequently, we did not perform a qualitative linguistic analysis of the individual framework steps, such as students’ specific prompt engineering strategies, cross‐referencing behaviors, or the discourse within the concluding seminar. Future research should incorporate process‐oriented qualitative evaluations to understand how individual interactions with AI directly mediate learning outcomes. No topic‐specific preintervention assessment was performed, so the quiz results represent postlearning group differences rather than demonstrated within‐student improvement. Finally, the cross‐context analyses were exploratory and based on self‐reported attitudes. The observed associations should therefore be regarded as hypothesis‐generating rather than evidence of causality, behavioral transfer, or clinical competence.

## 5. Conclusions

The novelty of this analysis lies in examining conceptually matched attitudes across two connected contexts: students using AI for their own learning and future clinicians responding to patients who have used AI before consultation. The results suggest that responsible engagement with AI may provide a link between these settings, particularly when openness to AI is accompanied by awareness of its risks and by professional respect for hypothetical AI‐informed patients. However, because the analysis was exploratory, based on a modest sample, and involved multiple conceptually selected comparisons, the significant associations should be interpreted as hypothesis‐generating rather than confirmatory.

For health‐professions education, the practical implication is that curricula should address both sides of AI use. Students need structured opportunities to use AI critically during learning, while also practicing how to verify, contextualize, and communicate about AI‐generated health information in patient encounters. The present findings support integrating these domains within a common educational framework, while retaining separate teaching and assessment for patient‐centered communication and clinical judgment.

## Author Contributions

Conceptualization, Jelena Roganović; methodology, Milena Barać and Igor Končar; validation, Jelena Roganović, Milena Barać, and Igor Končar; formal analysis, Jelena Roganović and Milena Barać; investigation, Igor Končar; writing–original draft preparation, Milena Barać, Igor Končar, and Jelena Roganović; writing–review and editing, Jelena Roganović.

## Funding

This research was funded by the Ministry of Education, Science and Technological Development of the Republic of Serbia (Grant No. 451‐03‐34/2026‐03/200129).

## Disclosure

All authors have read and agreed to the published version of the manuscript.

## Ethics Statement

The study was conducted in accordance with the Declaration of Helsinki and approved by the Ethics Committee of the School of Dental Medicine, approval No. 36/50, 2023.

## Consent

Informed consent was obtained from all subjects involved in the study.

## Conflicts of Interest

The authors declare no conflicts of interest.

## Data Availability

The data that support the findings of this study are available from the corresponding author upon reasonable request.
